# Comparative Genomics of the Extreme Acidophile *Acidithiobacillus thiooxidans* Reveals Intraspecific Divergence and Niche Adaptation

**DOI:** 10.3390/ijms17081355

**Published:** 2016-08-19

**Authors:** Xian Zhang, Xue Feng, Jiemeng Tao, Liyuan Ma, Yunhua Xiao, Yili Liang, Xueduan Liu, Huaqun Yin

**Affiliations:** 1 School of Minerals Processing and Bioengineering, Central South University, Changsha 410083, China; zixuange2010@126.com (X.Z.); fengxue@csu.edu.cn (X.F.); taojiemeng@csu.edu.cn (J.T.); maliyuan2008@csu.edu.cn (L.M.); huazipiaoling.123@163.com (Y.X.); liangyili@hotmail.com (Y.L.); xueduanliu@yahoo.com (X.L.); 2 Key Laboratory of Biometallurgy of Ministry of Education, Central South University, Changsha 410083, China

**Keywords:** *Acidithiobacillus thiooxidans*, comparative genomics, intraspecific diversity, niche adaptation

## Abstract

*Acidithiobacillus thiooxidans* known for its ubiquity in diverse acidic and sulfur-bearing environments worldwide was used as the research subject in this study. To explore the genomic fluidity and intraspecific diversity of *Acidithiobacillus thiooxidans* (*A. thiooxidans*) species, comparative genomics based on nine draft genomes was performed. Phylogenomic scrutiny provided first insights into the multiple groupings of these strains, suggesting that genetic diversity might be potentially correlated with their geographic distribution as well as geochemical conditions. While these strains shared a large number of common genes, they displayed differences in gene content. Functional assignment indicated that the core genome was essential for microbial basic activities such as energy acquisition and uptake of nutrients, whereas the accessory genome was thought to be involved in niche adaptation. Comprehensive analysis of their predicted central metabolism revealed that few differences were observed among these strains. Further analyses showed evidences of relevance between environmental conditions and genomic diversification. Furthermore, a diverse pool of mobile genetic elements including insertion sequences and genomic islands in all *A. thiooxidans* strains probably demonstrated the frequent genetic flow (such as lateral gene transfer) in the extremely acidic environments. From another perspective, these elements might endow *A. thiooxidans* species with capacities to withstand the chemical constraints of their natural habitats. Taken together, our findings bring some valuable data to better understand the genomic diversity and econiche adaptation within *A. thiooxidans* strains.

## 1. Introduction

Extraordinarily extreme environments [1,2,3] are the habitats for extremophiles, although they were previously thought of as almost insurmountable physical and chemical barriers to life [4]. These extreme environmental conditions are inhospitable to the growth of most life [5,6]. However, acidophilic microorganisms, especially prokaryotic acidophiles (eubacteria and archaea), are considerably diverse in natural and man-made acidic environments (pH < 3) [7]. Thus, it is of great interest to identify the potential mechanisms that ensure microorganisms survive and proliferate in these extreme environments.

With the advance of high-throughput sequencing, numerous genomes derived from a wide range of organisms were sequenced continuously, thereby fueling the development of comparative genomics [8]. In contrast to standard genetic researches, which have inherent limits to elucidate the hereditary traits of species, acquisition of genomic sequences generated by high-throughput sequencing provides plentiful gene contents and further enables future studies to explore the primary issues that investigators are interested in. Acidophiles such as *Acidithiobacillus*, *Sulfobacillus*, and *Leptospirillum* were isolated from the extremely acidic environments, and their sequenced genomes were used for comparative survey [6,9,10]. Nevertheless, limited information is currently known about intraspecific variability of acidophiles at the genome level. Comparative genomics determining genomic differences among multiple strains of individual species can unravel the extent of intraspecific diversity [11]. The gene repertoire represented across all strains reveals the genomic diversity of a species, i.e., pan-genome, which consists of “core genome” (common genes to all strains of a species) and “dispensable or accessory genome” (genes shared by some but not all strains of the species as well as strain-specific genes) [12]. The core genome includes all common genes that are essential for its basic lifestyle and major phenotypic traits, while dispensable genome confers selective advantages such as niche adaptation, antibiotic resistance, and colonization of new hosts [12,13]. However, it remains unclear whether these intriguing findings concerning the genomic analyses of other organisms could be applied to acidophiles isolated from the harsh environments that are physico-chemically and ecologically distinct from the “normal” environments.

*Acidithiobacillus*, the acidophilic and obligately chemolithoautotrophic bacterium, is widely found in various acidic environments worldwide [7,14]. *Acidithiobacillus thiooxidans* (*A. thiooxidans*) is an aerobic mesophilic microorganism belonging to the genus *Acidithiobacillus* and is considered to play an important role in industrial bioleaching [10]. Until recently, three genomes of *A. thiooxidans* strains including ATCC 19377, A01, and Licanantay from various habitats (Table 1) have been sequenced and submitted to National Center for Biotechnology Information (NCBI) [15,16,17]. When the draft genome sequences were released, numerous genes were annotated and public, thus providing a wealth of valuable information. As a result, much research could be invested to identify novel insights into the genotypic characteristics.

In this study, six new genomic DNA of *A. thiooxidans* strains isolated from different acidic environments in China (Table 1) were extracted and sequenced. Together with three aforementioned genomes publicly available in the GenBank database, a global genomic comparison was executed. Our work showed a data-driven approach to elucidate the similarities and differences among *A. thiooxidans* genomes, aiming to explore the genetic diversity and niche adaptation within *A. thiooxidans* strains.

## 2. Results and Discussion

### 2.1. General Features of Acidithiobacillus thiooxidans (A. thiooxidans) Genomes

Six new genomic DNA were subjected to Illumina MiSeq sequencing platform, and an average of 240 Mb raw reads (short DNA sequences) in each genome was yielded. After quality control using NGS QC Toolkit, high quality (HQ) reads (85.3% to 87.80%) were retained for subsequent analyses. All HQ reads aforementioned were used for sequence assembly, and an in-house Perl script was then employed to filter the assembled sequences under 200 bp, resulting in the draft genome assemblies. Genome characteristics were summarized in Table S1. Of note, the draft genome of *A. thiooxidans* ATCC 19377 sequenced previously [15] was much smaller than these of the others, preliminarily inferring that the low-coverage genome sequencing (9.6-fold) might contribute to the missing of large fragments genomic DNA. Nevertheless, high quality and completeness of genome assemblies was acquired in this strain (Table S1). Thus, pan-genome analysis could be reliable as the high quality of genome completeness was estimated in all strains.

**Figure 1:**
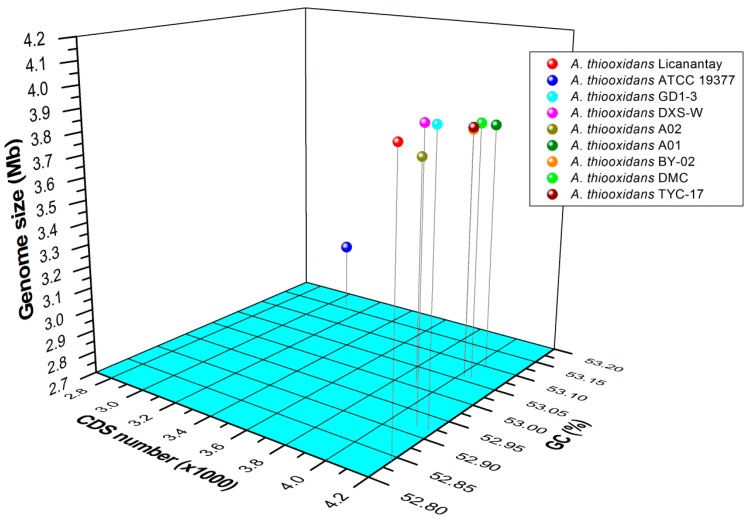
Three-dimensional plots of genome size, coding sequence (CDS) number, and GC content of the nine *Acidithiobacillus thiooxidans* (*A. thiooxidans*) strains sequenced in this study. The available genomes from strains Licanantay, ATCC 19377, and A01 were acquired from the public database, and the others were sequenced in this study.

As listed in Table S1, total genome sizes varied among all strains (3.02 to 3.95 Mb). As stated by Nuñez et al. [18], genome size in prokaryotes is related to metabolic diversity, effective population size, regulatory complexity, and horizontal transfer rates. And larger genomes might have a high adaptive plasticity compared to smaller genomes [17]. Previous studies showed that the genome of *A. ferrooxidans* DSM 16786 isolated from mining processes [19] was much larger than that of *A. ferrooxidans* ATCC 23270, which was from the bituminous effluent of coal mine [17]. Likewise, the larger genome size of *A. thiooxidans* strains GD1-3, DXS-W, and Licanantay obtained from copper mining implied that putative gene acquisition might enable them to adapt to the high concentration of metals in these metal mines. Also, the number of putative coding sequence (CDS) (3087 to 4200) and mean mole percentage GC content (52.84% to 53.17%) varied among all sequenced genomes (Figure 1 and Table S1). There is slight difference about mean percentage GC content among strains, while the number of CDS varied greatly. The CDS counts of strain Licanantay (4200) and GD1-3 (4171) were slightly more than those of the others.

### 2.2. Pan-Genome Analysis of A. thiooxidans Strains

**Figure 2:**
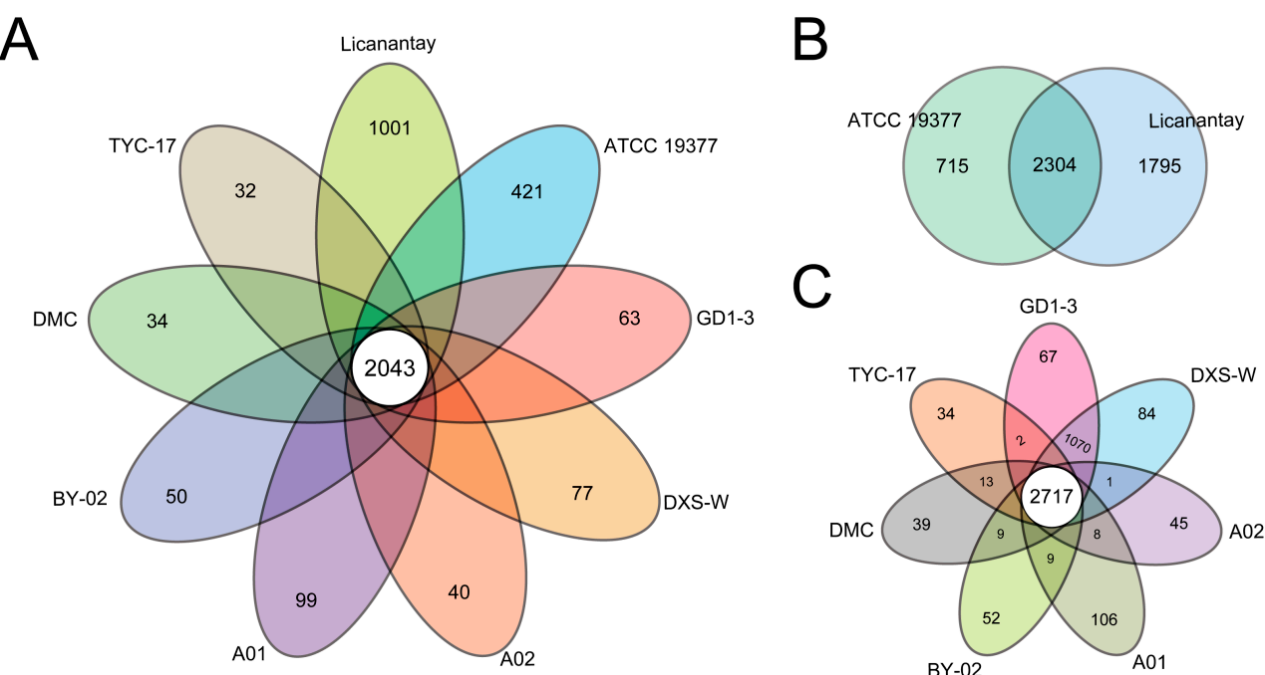
The pan-genome of *Acidithiobacillus thiooxidans* strains. The flower plots and Venn diagram demonstrate the number of shared, accessory and strain-specific genes among *A. thiooxidans* strains. Each strain was represented by an oval or circle that was colored. (**A**) Flower plot showing the numbers (in the petals) correspond to the unique genes of each strains, and the number of core genome common to all *A. thiooxidans* strains (in the center); (**B**) Venn diagram showing the numbers of unique genes and core orthologous genes between *A. thiooxidans* ATCC 19377 and Licanantay; (**C**) flower plot showing the numbers of CDSs among all *A. thiooxidans* strains in this study except for ATCC 19377 and Licanantay.

To understand the pan-genome of *A. thiooxidans* more deeply, 7186 protein CDSs obtained from the six newly sequenced genomes plus three available genomes from the public database were clustered using the program PanOCT with a 50% sequence identity cut-off. Herein, 2043 (28.31%) orthologs were identified as the *A. thiooxidans* core genome, and the remaining variable genes were defined as the accessory genome of *A. thiooxidans* (Figure 2A). In particular, our results showed that Licanantay had the largest number of unique genes (1001), followed by ATCC 19377 (421). These were similar to what Travisany et al. [17] reported previously. In their study, comparative analysis showed that strain-specific genes in the Licanantay might be involved in adaptation to its specific biomining environment and several genetic motility genes likely acquired by horizontal gene transfer in mining environments.

Considering the closely relation between Licanantay and ATCC 19377 [17], we further identified their shared genes. 2304 orthologous genes were present, and the unique genes in Licanantay and ATCC 19377 were 1795 and 715, respectively (Figure 2B), further indicating that Licanantay with much more strain-specific genes had the advantage to adapt the environmental conditions. Additionally, core orthologous genes and unique genes within seven other strains were examined. 2717 orthologs were identified, and the number of strain-specific genes in each strain varied from 34 to 106 (Figure 2C). In particular, GD1-3 and DXS-W shared a large number of genes (1070) only between the two of them, suggesting a closely correlation with each other.

### 2.3. Phylogenomic Tree Based on Core Genome

**Figure 3:**
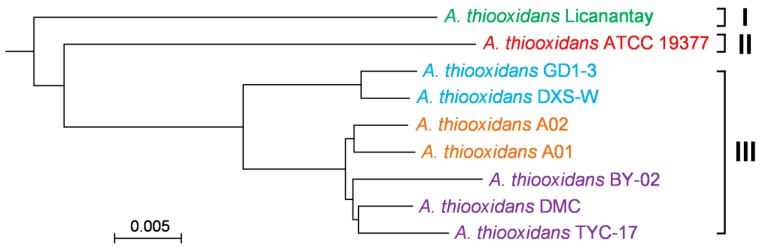
Phylogenomic tree of sequenced *Acidithiobacillus thiooxidans* strains based on their core genome. These strains from various geographic origins were clustered into three distinct classes. Classe I represents strain Licanantay isolated from Kimmeridge clay, class II represents strain ATCC 19377 from Chilean copper mine, and class III represents certain strains isolated from different acidic envornments in China.

Since the 16S rRNA gene sequences among each pair of *A. thiooxidans* strains are highly similar, we could not assess the phylogenetic distance between strains using these sequences alone. In this study, a phylogenomic tree based on their core genome (Figure 3) showed that nine strains were apparently divided into three main groupings. As depicted in Figure 3, *A. thiooxidans* Licanantay was genetically distinct from other strains included in this study, and strains GD1-3, DXS-W, A02, A01, BY-02, DMC, and TYC-17 were closely related to each other. In fact, the strains ATCC 19377 and Licanantay were originally isolated from Kimmeridge clay and Chilean copper mine respectively [15,17], and the others from various acidic environments in China (Table 1). Thus, a hypothesis was proposed that hereditary difference might be related to the geographic distribution.

Further inspection showed that these six strains from China were classified into three clusters. As reported by Douillard et al. [20], phylogenomic analysis showed that multiple groupings of *Lactobacillus rhamnosus* partly be related to their ecological niches. In our study, *A. thiooxidans* strains GD1-3 and DXS-W were isolated from the similar environments, and strains A01 and A02 from the same sampling points. While strains BY-02 and TYC-17 were isolated from copper mine, and DMC from coal heap drainage, these three strains gathered in a cluster. Unfortunately, the detailed geochemical conditions of these six bacteria, at that time, were not measured. Thus, the limited samples and experimental data restricted our further analysis to determine whether genetic difference was correlated with the geochemical characteristics in these acidic environments.

### 2.4. Functional Features of the Pan-Genome

**Figure 4:**
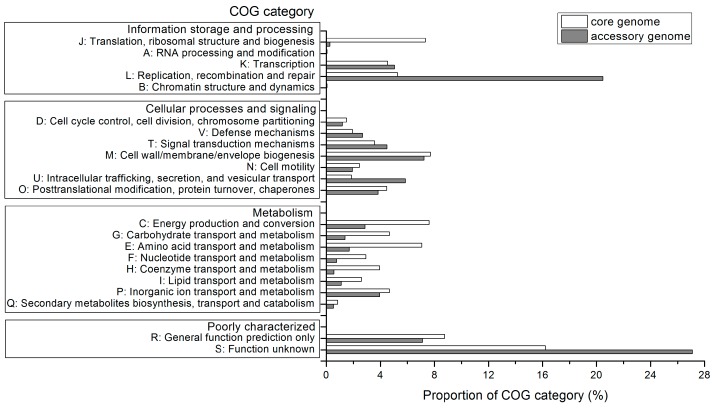
Distribution of core and flexible genes based on Clusters of Orthologous Groups (COG) category in *A. thiooxidans* strains. Only orthologous genes assigned to COG category were used for analysis.

To identify possible intraspecific diversification in functions, the functional annotation of core genome and accessory genome were performed against the specialized database Clusters of Orthologous Groups (COG). As shown in Figure 4, the abundances of metabolism-related genes assigned to COG categories (C) (energy production and conversion), (E) (amino acid transport and metabolism), (G) (carbohydrate transport and metabolism), (F) (nucleotide transport and metabolism), (H) (coenzyme transport and metabolism) and (I) (lipid transport and metabolism) in the core genome were greater in these *A. thiooxidans* strains compared to those in the accessory genome. These findings were reasonable given that these shared genes were involved in microbial basic activities, which might support the view that core genome was essential for basic lifestyle of species [12,13]. Additionally, the core genome was highly enriched in COG category (J) (translation, ribosomal structure and biogenesis) relative to accessory genome. These features were similar to what other researchers have been found in their respective pan-genome analyses [21,22]. Especially, the core genome in all strains was commonly enriched in the COG category (M) (cell wall/membrane/envelope biogenesis). We interpreted this as an indication that *A. thiooxidans* strains inhabiting the acidic environments shared distinctive structural and functional properties to maintain a stable pH gradient, as specialized cellular structures were regarded to be important for acidophile pH homeostasis [23].

In contrast, the accessory genome among *A. thiooxidans* strains consisted of putative 5143 CDSs, and COG class assignment revealed the abundant CDSs were involved in replication, recombination and repair (COG category (L); Figure 4). Considering that the high concentration of toxic substances such as heavy metals in these acidic environments [7,24], and the high level of heavy metals concentration might cause a high rate of DNA injury [25], it appears to be reasonable that there were the abundant genes in the accessory genome assigned to COG category (L), which might be related to niche adaptation. This finding was in line with previous comparative genomics showing that the accessory genome of 48 strains of sinorhizobia *Sinorhizobium* comprising five genospecies might be related to the different strategies to interact with diverse host plant and soil environments [21]. Also, comparative analysis of *Klebsiella pneumoniae* Kp13 showed that genomic plasticity occurring at multiple hierarchical levels might play a role of the lifestyle [26]. Besides, both core genome and accessory genome had high proportion of genes in COG categories (R) (general prediction only) and (S) (function unknown). The amino acid sequences associated with these CDSs which lacked a functional assignment were then chosen for functional annotation against the NCBI-NR database (*E*-value ≤ 10^−5^). Results indicated that various proportions of CDSs (20.55%~74.03%) were hypothetical proteins, and the others were annotated as proteins with known function (Table S2). For these functional proteins, we found most CDS showing hits with *A. thiooxidans*. We emphasized the reasonability of these findings that CDS in COG categories (R) and (S) could be re-annotated as proteins with known function in our study, due to the continuously updated database.

We also performed a Blast search against the NCBI-nr database using the protein sequences related to strain-specific genes. Similar to several other comparative genomic analyses [27,28,29], a large number of CDS were annotated as hypothetical proteins. Furthermore, most of them were not assigned to the COG category (Figure S1A). Especially, we further inspected the non-shared CDS from *A. thiooxidans* Licanantay and ATCC 19377 considering that the numbers were larger compared to those in other strains. The most abundant strain-specific CDS were assigned into COG category (L) and (M) (Figure S1B), which was consistent with previous study [17]. These strain-specific genes might confer them some advantages to adapt to the environmental conditions.

### 2.5. Identification of Metabolic Traits and Management Strategies to Environmental Stress

#### 2.5.1. Feature of Central Metabolism

**Figure 5:**
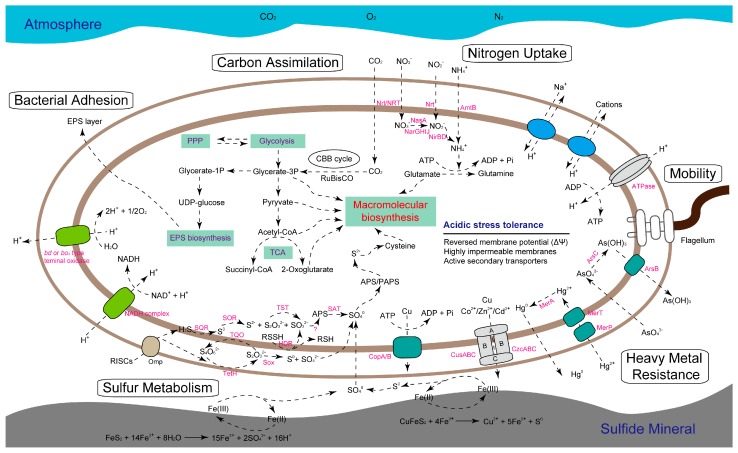
Schematic diagram depicting the predicted central metabolism and potential management strategies to environmental stress of *A. thiooxidans* strains. Herein, several genes in *A. thiooxidans* ATCC 19377 were absent. Included were genes involved in nitrate reduction, genes encoding sulfur oxygenase reductase and nitrate/nitrite transporter. More details for genes/enzymes involved in central metabolism and environmental adaptation were presented in Supplementary Table S3.

The assignment of CDSs to the COG classification revealed inspection concerning the metabolic traits of *A. thiooxidans* strains, highlighting the high abundance of metabolic profiles in the core genome (Figure 4). In this study, the number of assigned CDSs involved in central metabolism, including carbon assimilation, nitrogen uptake, and sulfur metabolism, was discussed (Table S3). Subsequently, the metabolic potentials of *A. thiooxidans* were reconstructed and compared to each other in order to determine the shared or strain-specific metabolic feature. As depicted in Figure 5, all strains have the ability to fix carbon atmospheric CO_2_ via Calvin Benson Bassham cycle. In particular, *A. thiooxidans* harbors a gene cluster potentially encoding carbon dioxide-concentrating protein, carboxysome shell protein, carboxysomal shell carbonic anhydrase, and ribulose-1,5-bisphosphate carboxylase/oxygenase, allowing a higher efficiency for CO_2_ fixation within the carboxysome [30]. The product 3-phosphoglycerate (G3P) generated in the process of CO_2_ fixation was predicted to be converted to be the precursors for the macromolecular biosynthesis such as amino acids, fatty acids. Particularly, the conversion of G3P was expected to result in the formation of UDP-glucose, a major precursor for biosynthesis of extracellular polysaccharide [31]. The latter could mediate bacterial adhesion and biofilm formation [32], and provide a reaction space between bacterial cell and mineral surface, thus increasing the dissolution of metal sulfides [32,33].

Unlike closely related *A. ferrooxidans*, which has the set of genes required for N_2_ fixation via nitrogenase [34], *A. thiooxidans* strains shared the potential to assimilate the nitrate, nitrite, as well as ammonium as nitrogen sources necessary for their growth (Figure 5). The accumulated ammonium derived from the reduction of nitrate and nitrite, or from the uptake of extracellular ammonium via Amt family transporter, would enter into the biosynthesis of glutamate and other vital compounds. Results showed that all strains were found to harbor a full suite of genes involved in nitrogen metabolism except for strain ATCC 19377. For example, several genes associated with assimilatory and assimilatory nitrate reduction were not identified in its genome (Table S3). Also, genes encoding transporters for nitrate or nitrite were absent. One possible explanation was that strain ATCC 19377 could take up environmental ammonium as its sole pathway for the acquisition of nitrogen source.

Referred to the well-studied models for sulfur oxidation in *A. thiooxidans* species [10,35], a hypothetical model for sulfur oxidation system and electron transportation was reconstructed in our study (Figure 5). In this model, a series of complicated enzymatic reactions were performed by various enzymes with distinguished features in specific cellular position. The *sor* gene encoding sulfur oxygenase reductase, an enzyme directing the disproportionation reaction of sulfur to generate sulfide, thiosulfate, as well as sulfate [36], was absent in strain ATCC 19377. It seems that horizontal gene transfer might occur in this strain. However, it remains to be further validated.

Our results showed that the vast majority of homologous genes were identified by aligning against the public database as well as reported sequences in other literatures, although there were few exceptions such as genes involved in nitrate reduction in strain ATCC 19377. Taken together, we proposed that there were few intraspecific differences with respect to metabolic pathways, at least central metabolism.

#### 2.5.2. Predicted Stress Tolerance Mechanisms

The comparison of metabolic profiles of *A. thiooxidans* strains was extended to potential mechanisms to respond to environmental stress, including acidic pH, high concentration of toxic substrates such as heavy metal ion and organic compounds (not shown in Figure 5). Also, cell mobility was taken into account. In this study, all strains shared a core set of genes potentially related to stress management. Like most other acidophiles, *A. thiooxidans* species highly relied on intracellular pH homeostasis, allowing it to grow at distinct ranges of pH values [37]. Numerous genes assigned to COG category (N) (cell mobility) were predicted to be involved in flagella formation (Table S3). The presence of these putative genes indicated that all *A. thiooxidans* strains had the capacity to actively move in aquatic habitats. Additionally, the identification of several gene clusters related to the resistance of heavy metals including arsenic, mercury, cadmium, and copper suggested that all strains might employ various systems to cope with high metal ion concentrations. As for organic solvents such as Lix984n, an organic extractant used for metal extraction in industrial operation [38], a putative gene cluster *acrAB*-*tolC* encoding a member of resistance-nodulation-cell division (RND) family protein was predicted to potentially transfer this substrate [39]. Additionally, *A. thiooxidans* strains were found to harbor a complete six-gene cluster for ATP binding cassette (ABC) transport system involved in toluene resistance (Table S3). In conclusion, all strains observed in this study appear to exhibit similar strategies to cope with the chemical constraints of their natural habitats.

### 2.6. Gene Turnover Analysis

Similar to earlier study in the closely related *Acidithiobacillus caldus* [40], prediction and classification of transposases using ISFinder showed that 3.41%~4.54% of the predicted CDSs of *A. thiooxidans* strains encoded transposases belonging to 25 different insertion sequence (IS) families (Table 2). The most abundant IS families in all strains were ISL3. High similarity regarding IS type were observed in all *A. thiooxidans* strains, however, closer inspection demonstrated several differences. For instance, *A. thiooxidans* strains GD1-3, DXS-W, and Licanantay with larger genome size had more transposases than the others. This finding might be reasonable considering that horizontal transfer was regarded as an evolutionary force to increase microbiological DNA content [41]. On the other hand, a frequent gene flow and genetic drift might endow species with adaptive capacity to the extreme econiche.

Besides, finding presented in this study revealed that many genomic island (GI) occurred in the *A. thiooxidans* population (Table 2). Further analyses suggested that several integrases or mobile genetic elements were presented in the predicted GI (not shown in Table 2), thereby indicating that these putative GI were likely acquired by horizontal gene transfer. As stated by Wu et al. [42], GI were highly related to the niche-specific adaptation. Thus, it was inferred that these GI might play a key role in adapting to specific lifestyles and environmental niches. In short, great frequency of genetic exchange might provide *A. thiooxidans* species with adaptive advantage in extremely acidic environments.

## 3. Materials and Methods

### 3.1. Bacterial Strains Used in This Study

We included a total of six strains of *A. thiooxidans* in this study, which were preserved in the acidophile culture collection maintained at Central South University, Changsha, China (Table 1). Each strain was cultivated in liquid 9K basic medium, including the following ingredients (grams per liter): (NH_4_)_2_SO_4_, 3.0; MgSO_4_·7H_2_O, 0.5; KCl, 0.1; Ca(NO_3_)_2_, 0.01; and K_2_HPO_4_, 0.5. Of this solution, elemental sulfur (autoclave-sterilized, 10g/L) was added as energy source. The culture temperature and shaking speed of rotary platform were 30 °C and 170 rpm, respectively. High-concentration cells (1~2 × 10^8^ cells/mL) were collected at the exponential growth phase (generally 3th day), and their genomic DNA were extracted using TIANamp Bacteria DNA Kit (TIANGEN, Beijing, China) following the manufacturer’s introductions.

### 3.2. Genome Sequencing and Bioinformatics Analyses

The genomes of six strains of *A. thiooxidans* were sequenced using the Illumina MiSeq platform for 2 × 150 bp paired-end sequencing (Illumina, Inc., San Diego, CA, USA). The raw reads were filtered using NGS QC Toolkit [43] with Phred 20 as a cutoff, and then paired-end read sequences with high-quality were assembled using velvet [44]. For each strain, several kmers were run and the best assembled result was chosen for subsequent analyses. Following the cleanup step, the contigs were further clustered and assembled de novo to obtain unigene sequences using iAssembler tool [45]. The genome completeness of each strain was estimated using the program CheckM [46]. Coding sequences (CDS) prediction was performed by MetaGeneAnnotator [47], and predicted genes were then functionally annotated via homology searching against the generalist databank (NCBI-NR) and specialised databases (COG and KEGG). The hidden Markov models for the protein domains were predicted via searching against the Pfam database [48]. The online platform tRNAscan-SE was used for the identifications of tRNA [49]. Additionally, insertion sequences and transposases were identified by Blastp against the ISFinder database [50] with manual inspection of search hits (*E*-value ≤ 10^−5^). The putative genomic islands were also predicted using the online platform IslandViewer 3 [51].

### 3.3. Pan-Genome Analysis

Gene annotations of assembled contigs of the six strains were performed through the RAST annotation server [52]. The GenBank files containing the genome information of *A. thiooxidans* strains were then downloaded. Additionally, three other available genome sequences from homologous strains including *A. thiooxidans* strains A01, ATCC 19377, and Licanantay were obtained from NCBI. For these nine strains, the corresponding protein sequences with Fasta format were extracted using in-house Perl scripts, and were then aligned using an all-versus-all BLASTP. Output files with the m8 BLAST format were used for the identification of single-copy orthologs using PanOCT program (50% identity cut-off; *E*-value ≤ 10^−5^) [53]. Finally, the shared and dispensable genes in all strains were functionally annotated as described above.

### 3.4. Phylogenomic and Phylogenetic Analyses

A core of ortholog genes from nine draft genomes of *A. thiooxidans* strains were extracted using in-house Perl script. These shared genes were then used to construct a genome-based and alignment-free phylogeny using the online platform CVTree3 with K-tuple length 6 [54]. Additionally, the genome of *Acidithiobacillus caldus* SM-1 was included as an out-group. Visualization for phylogenetic tree was performed using the sofeware MEGA v5.05 [55].

### 3.5. Data Deposition

These Whole Genome Shotgun projects of six *A. thiooxidans* strains have been deposited at the DDBJ/ENA/GenBank under the accession LWSC00000000 (GD1-3), LWRY00000000 (DXS-W), LWSA00000000 (A02), LWRZ00000000 (BY-02), LWSB00000000 (DMC), and LWSD00000000 (TYC-17). Additionally, the versions described in this paper are version LWSC01000000, LWRY01000000, LWSA01000000, LWRZ01000000, LWSB01000000, and LWSD01000000, respectively.

## 4. Conclusions

Comparative genomics provided useful information on the genetic and functional features of *A. thiooxidans* strains. Phylogeny based on their core genome revealed that genetic diversity was potentially related to the geographic distribution and geochemical conditions of their habitats. Functional assignment of common genes uncovered that abundant genes were involved in metabolism, such as COG categories (C), (E), and (G), compared to the accessory genome, indicating that these genes were necessary for the microbial basic activities. Comprehensive analysis further showed little intraspecific diversification with respect to their predicted metabolic profiles. Additionally, most genes belonging to the dispensable genome were assigned to the COG category (L) and (M), suggesting a correlation between accessory genome and niche adaptation. Also, a considerable diverse repertoire of mobile genetic elements including insertion sequences and genomic islands were widespread in the draft genomes of *A. thiooxidans* strains obtained from various geographic origins, indicating that gain and/or loss of these elements by transferring horizontally might greatly contribute to intraspecific divergence and adaptation to acidic econiches.

## Figures and Tables

**Table 1:** Strains of *Acidithiobacillus thiooxidans* used for comparison survey in this study.

Strain	Geographic Origin	Reference
Licanantay	Copper mine, Atacama, Chile	[17]
ATCC 19377	Kimmeridge clay, UK	[15]
GD1-3	Copper Mine, Shaoguan, Guangdong, China	This study
DXS-W	Copper Mine, Dongxiang Mountain, Hami, Xinjiang, China	This study
A02	Coal heap drainage, Pingxiang, Jiangxi, China	This study
A01	Coal dump, Pingxiang, Jiangxi, China	[16]
BY-02	Copper Mine, Baiyin, Gansu, China	This study
DMC	Coal heap drainage, Chenzhou, Hunan, China	This study
TYC-17	Copper Mine, Baiyin, Gansu, China	This study

**Table 2:** The prediction of mobile elements including insertion sequences (IS) and genomic island (GI) in all *A. thiooxidans* strains observed in this study.

A. The Putative Insertion Sequences									
IS Family	DXS-W	Licanantay	A01	ATCC 19377	GD1-3	DMC	A02	BY-02	TYC-17
IS110	9	11	9	4	8	9	9	9	**10**
IS1182	2	0	0	0	2	0	0	0	0
IS1380	3	2	0	0	3	0	0	0	0
IS1595	5	8	8	4	5	9	7	9	8
IS1634	2	1	3	9	1	3	3	3	3
IS200/IS605	6	15	4	3	5	4	2	4	4
IS21	6	8	**11**	2	6	8	9	9	9
IS256	1	8	2	**15**	1	3	3	2	2
IS3	**11**	12	4	1	10	6	5	5	5
IS30	1	2	0	3	1	2	2	2	2
IS4	7	6	3	**12**	7	9	7	7	7
IS481	8	1	2	1	8	2	2	1	2
IS5	4	4	5	5	4	6	5	5	6
IS51	0	2	0	2	0	0	0	0	0
IS605	1	0	1	0	1	1	1	1	1
IS607	4	4	1	0	4	4	1	4	1
IS630	**11**	**18**	6	9	**12**	**14**	**12**	**11**	9
IS66	**11**	0	1	0	9	2	1	1	1
IS91	**20**	**16**	**16**	8	**19**	**15**	**16**	**16**	**15**
ISAs1	1	2	1	0	1	1	0	1	0
ISAzo13	1	1	0	0	1	0	0	0	0
ISKra4	7	2	4	2	7	1	4	1	4
ISL3	**43**	**44**	**39**	**30**	**47**	**41**	**41**	**40**	**42**
ISNCY	2	1	1	0	2	1	2	2	2
Tn3	**21**	**16**	**17**	**16**	**20**	**19**	**19**	**20**	**21**
Total	187	184	138	126	184	160	151	153	154
**B. The Predicted Genomic Islands**									
**Strain**	**DXS-W**	**Licanantay**	**A01**	**ATCC 19377**	**GD1-3**	**DMC**	**A02**	**BY-02**	**TYC-17**
GI Number	65	54	39	36	56	53	51	50	44

The four most abundant IS families were highlighted in bold.

## Supplementary Materials

Supplementary materials can be found at www.mdpi.com/1422-0067/17/8/1355/s1.
