# Supplementary material for: Comparative Genomics of the Extreme Acidophile Acidithiobacillus thiooxidans Reveals Intraspecific Divergence and Niche Adaptation
Source: Int J Mol Sci. 2016 Aug 19;17(8):1355. doi: 10.3390/ijms17081355 (PMC5000751; doi:10.3390/ijms17081355)
Supplement: Supplementary file 1 [file ijms-17-01355-s001.zip › ijms-141532-Supplementary Materials/ijms-141532-Figure S1 for publish.pdf]

Supplementary Materials: Comparative Genomics of the Extreme Acidophile *Acidithiobacillus thiooxidans* Reveals Intraspecific Divergence and Niche Adaptation

Xian Zhang, Xue Feng, Jiemeng Tao, Liyuan Ma, Yunhua Xiao, Yili Liang, Xueduan Liu and Huaqun Yin

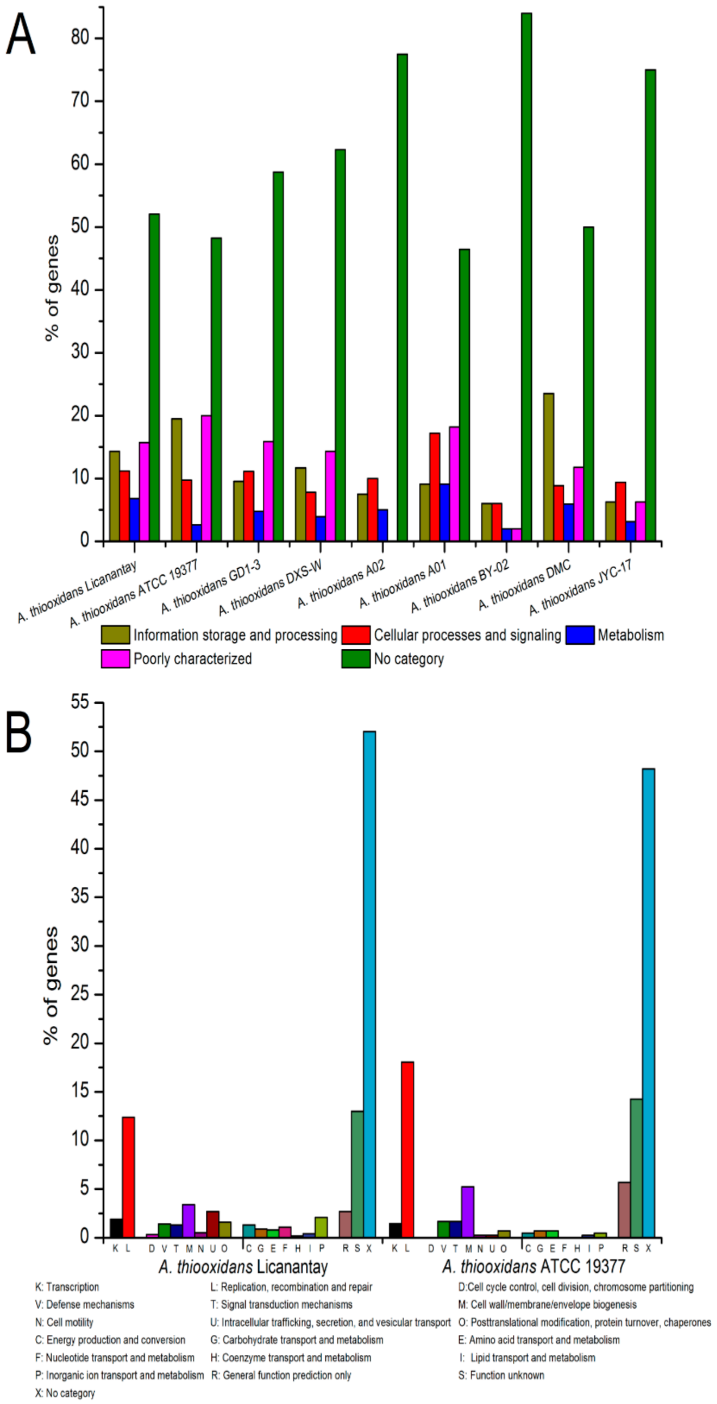

Figure S1. Classification of strain-specific genes in *A. thiooxidans* strains. Coding sequences (CDSs) were assigned to the Clusters of Orthologous Groups (COG) super-functional category (**A**) and the COG functional category (**B**).
